# The impacts of air pollution on maternal stress during pregnancy

**DOI:** 10.1038/srep40956

**Published:** 2017-01-18

**Authors:** Yanfen Lin, Leilei Zhou, Jian Xu, Zhongcheng Luo, Haidong Kan, Jinsong Zhang, Chonghuai Yan, Jun Zhang

**Affiliations:** 1 Xinhua Hospital, MOE-Shanghai Key Laboratory of Children’s Environmental Health, Department of Child and Adolescent Healthcare, Shanghai Institute for Pediatric Research, Shanghai Jiao Tong University School of Medicine, Shanghai, 200092 China; 2 School of Public Health, Key Laboratory of Public Health Safety of the Ministry of Education and Key Laboratory of Health Technology Assessment of the Ministry of Health, Fudan University, Shanghai, 200032 China; 3 Shanghai Key Laboratory of Atmospheric Particle Pollution and Prevention (LAP3), Fudan University, Shanghai, 200032 China

## Abstract

To investigate the association of air pollution with maternal stress during pregnancy, we enrolled 1,931 women during mid-to-late pregnancy in Shanghai in 2010. The “Life-Event Scale for Pregnant Women” and “Symptom-Checklist-90-Revised Scale” (SCL-90-R) were used to evaluate life event stress and emotional stress, respectively. Air pollution data were collected for each district where pregnant women lived during pregnancy. We associated ambient air pollution with stress scores using multivariable logistic regression models. After adjusting for relevant covariates, an interquartile-range (IQR) increase in sulphur-dioxide (SO_2_) (OR = 1.30, 95% CI: 1.11–1.52) and particulate-matter with an aerodynamic-diameter <10 μm (PM_10_) (OR = 1.16, 95% CI: 1.02–1.34) concentrations on the recruitment day, and in the 5-day moving average concentrations of nitrogen-dioxide (NO_2_) (OR = 1.34, 95% CI: 1.05–1.70) were associated with high Global-Severity-Indices (P75-P100) of the SCL-90-R. These associations were stronger among women bearing high levels (P25-P100) of air pollutants than among women experiencing low levels (P1-P25) of pollutants. The stronger associations and higher levels of pollutants were observed in the cool season than in the warm season. SO_2_ increases on the recruitment day were also associated with an increased risk of high depression scores (P75-P100). Our findings supported a dose-dependent association between air pollution and emotional stress during pregnancy.

As the largest developing country in the world, China has achieved rapid development in the past two decades. However, this economic development is accompanied by increased levels of air pollution, particularly in large cities. In Shanghai, one of the largest developed cities in China, officials have become aware of the issue of air pollution and have implemented various prevention measures. Nonetheless, air pollution may remain a threat to public health in Shanghai^1^,^2^.

In addition to the effects of air pollution on somatic disorders that have been extensively investigated, such as increased risks of pulmonary diseases^3^,^4^,^5^, cardiovascular diseases^6^,^7^, or mortality^8^,^9^,^10^, a few studies have focused on the association between ambient air pollution and human psychological health. Exposure to air pollution was found to be related to increased risks of suicide^11^, and depressive symptoms^12^. Air pollution was also reported to be linked with higher perceived stress levels in old men, particularly in cold months^13^. Studies also reported that short-term exposure to ambient air pollution was associated with increased emergency department visits because of depression or suicide attempts^14^,^15^,^16^.

The developing foetus is vulnerable to many adverse factors, including maternal stress during pregnancy. Previous studies have shown that excessive maternal stress during pregnancy could induce preterm birth^17^, low birth weight^18^, and adverse neurodevelopment outcomes in infants, including cognitive deficits and changes in temperamental traits^19^,^20^. However, until now, no evidence is available to show the association between air pollution and maternal stress during pregnancy. Previous studies also reported that the respiratory diseases or symptoms differed at different levels of air pollution^21^,^22^. Higher levels of air pollution may be more likely to induce oxidative stress, which played an important role in the development of depression^23^; therefore, we wondered whether the magnitude of the association between air pollution and maternal stress during pregnancy varied at different levels of air pollution. Additionally, the complications of pregnancy were related to maternal stress during pregnancy^24^,^25^, and air pollution was associated with the development of complications of pregnancy^26^. However, the modifying effects of complications of pregnancy on the association between air pollution and maternal stress during pregnancy remain unclear^27^,^28^.

Therefore, the present study was to evaluate the association between major air pollutants, including sulphur dioxide (SO_2_), nitrogen dioxide (NO_2_), and particulate-matter with an aerodynamic-diameter <10 μm (PM_10_) and maternal stress levels during pregnancy in Shanghai, China.

## Methods

### Study design

**Figure 1:**
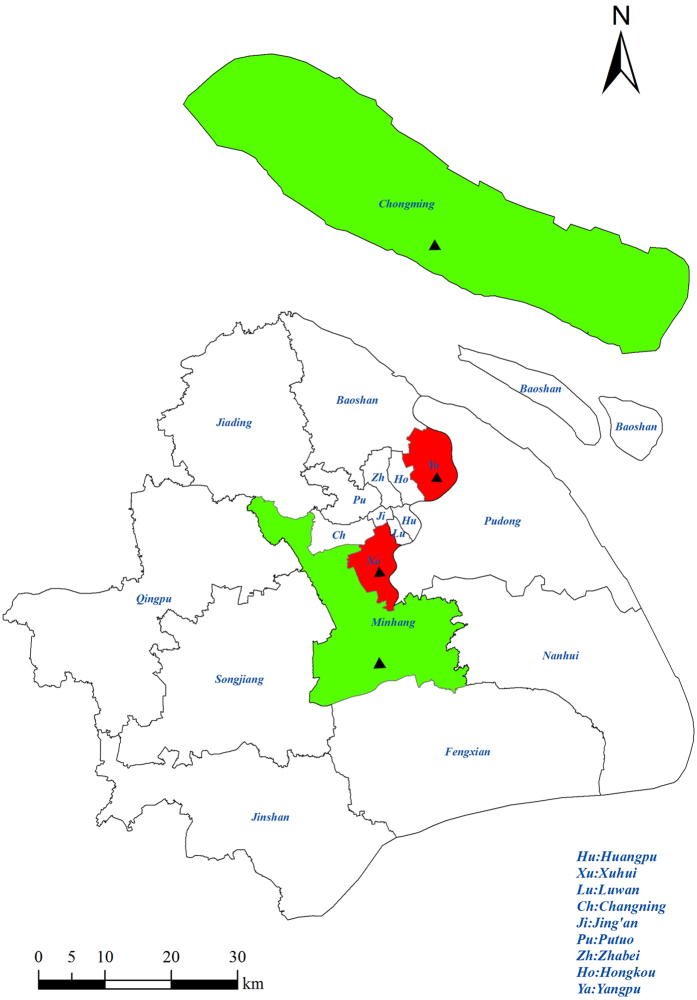
The map of Shanghai with the sampling locations marked. (The map was made by ArcGIS 10.0, URL link: http://www.esri.com/). A total of 1931 pregnant women living in the 19 districts of Shanghai were enrolled in 2010 in this study. The selected districts and hospitals were marked as above (hospitals were marked with triangle, the selected 2 urban districts were filled with red, and the selected 2 surburban districts were filled with green). The 9 urban districts: Huangpu, Luwan, Xuhui, Changning, Jingan, Putuo, Zhabei, Hongkou, Yangpu; The 10 suburban districts: Baoshan, Minhang, Jiading, Songjiang, Qingpu, Fengxian, Jinshan, Pudong, Nanhui, Chongming.

This study evaluated data from 1,931 pregnant women who regularly visited 4 prenatal-care clinics for antenatal examination from February to October, 2010 in Shanghai. These women were recruited using stratified-cluster sampling. According to the 2010 Census, there were a total of 19 districts in Shanghai. The study districts were sampled among all districts in Shanghai. We randomly selected 2 districts (Minhang and Chongming) from the 10 suburban districts and 2 districts (Yangpu and Xuhui) from the 9 urban districts (the map of the sampling locations was shown in Fig. 1). Within each selected district, the largest maternity hospital was chosen. Therefore, 2 suburban hospitals (Xinhua Hospital Chongming branch and Minhang Maternal and Child Health Hospital) and 2 urban hospitals (International Peace Maternity and Child Health Hospital and Xinhua Hospital) were finally included, covering the north, south and central areas of Shanghai. These 4 hospitals were among the largest maternity hospitals in Shanghai, serving pregnant women from not only the districts where the hospitals were located but also from other districts. The study women were finally from all the districts of Shanghai (Fig. 1)^29^.

We conducted an in-person interview to collect information on the women’s socio-demographic characteristics and on their diseases and pregnancy histories. Women with “mental disabilities” were excluded according to their maternity medical record kept in prenatal care clinics^29^. Special stress scales were used to assess the women’s life event stress levels and emotional stress levels during pregnancy. Daily air quality data were collected for individual women.

We obtained written informed consent from all participants, and the study protocol was approved by the Medical Ethics Committee of Xinhua Hospital affiliated with the Shanghai Jiao Tong University School of Medicine. All the methods applied in the study were carried out in accordance with the approved guidelines.

### Measures of maternal stress

Trained staff conducted face-to-face interviews to evaluate maternal stress during pregnancy using the Life Event Scale for Pregnant Women (LESPW) and the Symptom Checklist-90-Revised Scale (SCL-90-R).

The LESPW is a self-rating scale used to assess perceived stress levels during pregnancy^30^. This scale consists of 53 items on family, work, learning, and social relationship-related problems, and the events are weighted to calculate the LESPW total score. The LESPW is a valid and reliable tool that has been widely used to assess life event stress levels during pregnancy in China^31^. A higher LESPW score indicates a higher level of life event stress during pregnancy.

The SCL-90-R is a self-reported inventory used to assess emotional stress or psychosocial distress^32^. Each individual symptom is rated as 1 (no distress), 2 (slightly distressed), 3 (moderately distressed), 4 (quite a bit of distress), and 5 (extremely distressed). The scale consists of 90 distress items to assess symptoms in 9 subscales, including somatization, obsessive-compulsive, interpersonal sensitivity, depression, anxiety, hostility, phobic anxiety, paranoid ideation, and psychoticism. The SCL-90-R is a scale with good reliability and validity, and has been widely used in China^33^. We used the mean score of all items (90 items), namely the Global Severity Index (GSI) (GSI score = SCL-90-R total score/90), to represent the overall level of emotional stress, and we used the mean score of the items belonging to each subscale (total score of the subscale/the number of the items of the subscale) to represent the degree of the psychiatric symptom associated with the subscale. The GSI is a good indicator of the severity of general psychiatric symptoms. A higher GSI score indicates a higher level of emotional stress, and a higher subscale score suggests a higher level of the specific psychiatric symptom^32^.

### Air pollution variables

We obtained daily air pollution data (including daily average SO_2_, NO_2_, and PM_10_ concentrations) from the Shanghai Environmental Monitoring Centre based on the districts where the participating pregnant women lived during pregnancy. A 3-day moving average concentration (the recruitment day and the previous 2 days), a 6-day moving average concentration (the recruitment day and the previous 5 days), an 8-day moving average concentration (the recruitment day and the previous 7 days), and a 15-day moving average concentration (the recruitment day and the previous 14 days) of air pollution levels (including SO_2_, NO_2_, and PM_10_) were calculated for each woman according to the daily average concentration of each pollutant. Acute and delayed effects of air pollutants on maternal stress were estimated using the following moving average lag structure: 0 day (air pollution levels on the recruitment day), 0–2 days (3-day moving average concentration), 0–5 days (6-day moving average concentration), 0–7 days (8-day moving average concentration), and 0–14 days (15-day moving average concentration).

### Confounders

To control for the potential confounding effects of weather conditions, weather data during the study period, including daily average temperature, relative humidity, and wind speed were obtained from the Shanghai Meteorological Bureau. Other covariates, including maternal age, education, occupation, family monthly income, type of family structure, complications of pregnancy, and abortion history were collected through questionnaires and in-person interviews. Confounders were selected based on the following commonly used criteria: the variable was associated with the main predictor (air pollution) or the dependent variable (maternal stress level), and it was not in the causal pathway between the outcome and the main predictor.

### Statistical analysis

We first examined the socio-demographic characteristics of the 1,931 participants and the overall characteristics of the air quality and weather. Spearman correlation was used to examine the interrelationship between air pollutants and weather variables. Multivariate binary logistic regression analyses were used to evaluate the relationships between each air pollutant and maternal stress levels during pregnancy. Because the GSI, depression, and anxiety scores of the SCL-90-R and LESPW data showed non-normal distributions and there were no accepted reference ranges for these scores among pregnant women, we performed sensitivity analyses to determine the cut-off values for the definitions of high levels of maternal GSI/depression/anxiety/LESPW. We found that, for the GSI or depression scores, every 5 percent increase in the cut-off values of GSI or depression scores from the 75th percentile to the 95th percentile didn’t significantly change the direction and strength of the associations between air pollution and maternal stress levels, however, every 5 percent decrease in the cut-off values of GSI or depression scores from the 75th percentile to the 50th percentile changed the strength of the associations (from significant positive associations to non-significant associations). In addition, the 75th percentile was frequently used as the cut-off value in previous studies. Therefore, high levels of GSI/depression/anxiety/LESPW were defined as GSI/depression/anxiety/LESPW scores ranging from P75-P100. The estimated effect of an interquartile range (IQR) increase in the concentration of each air pollutant (including SO_2_, NO_2_, and PM_10_) was expressed as the increased risk of maternal emotional stress/life event stress^12^. *P* < 0.05 (2-tailed) was considered statistically significant.

To investigate whether the magnitude of the association between each air pollutant and maternal stress during pregnancy differed according to the levels of air pollutants, air pollution was categorized to simplify the interpretation. We estimated the relationship between each air pollutant and maternal stress separately within each quartile of air pollutant levels. We observed that for the highest 3 quartiles of air pollutant concentrations, the increase in each air pollutant level was significantly associated with an increased risk of high emotional stress (P75-P100), however in the lowest quartile, the increase in each air pollutant level was non-significantly associated with the risk of high emotional stress. Therefore, the high level of air pollution was defined as “the average concentrations of the air pollutants (including SO_2_, NO_2_ and PM_10_) among P25-P100”, and the low level of air pollution was defined as “the air pollutant concentrations (including SO_2_, NO_2_ and PM_10_) among P1-P25”.

Based on the lag structure that provided the best fit to the data, we further analysed the effects of air pollution separately for the warm season (from May to October, when the daily average temperatures ranged from 15.3 to 34.1 °C and the mean temperature was 24.7 °C) and the cool season (from November to the next April, when the daily average temperatures ranged from −0.6 to 17.9 °C and the mean temperature was 12.1 °C)^34^.

To investigate whether complications of pregnancy modified the association between air pollution and maternal stress levels, the participants were classified into two categories (yes or no) based on whether they had complications of pregnancy or not, including gestational hypertension, gestational diabetes mellitus, anemia, polyhydramnios, oligohydramnios, intrahepatic cholestasis of pregnancy, hypohepatia and heart diseases, and the modifying effects were examined in the association between air pollution and maternal stress. We combined women with different kinds of complications mentioned above as the “complication” (yes) group, and women without any kind of complications as the “control” (no) group. In addition, because of the limited sample size for each type of complication except for anemia [the sample size of women with anemia was 129, and the sample sizes of women with other kinds of complications of pregnancy were all ≤65 (≤3.4% of our study population)], we only additionally estimated the modifying effect of anemia on the association between air pollution and maternal stress.

In order to examine the independent association of a certain pollutant after adjusting for the potential confounding effects caused by other pollutants, two- and three-pollutant models were used in our analyses.

The association of levels of air pollutants with depression and anxiety symptoms was estimated using a multivariate binary logistic regression model. We expressed the estimated effects of an IQR increase in the concentration of each pollutant (including SO_2_, NO_2_, and PM_10_) as the increased risks of depression and anxiety symptoms.

All analyses were performed using Empower (R) (www.empowerstats.com, X&Y Solutions, Inc., Boston, MA, USA) and R (http://www.R-project.org).

## Results

The average age of these 1,931 pregnant women was 28.0 years old, and most of the women were well-educated (58.6% of the women finished their college/university or postgraduate education). The proportion of the women who had various types of complications of pregnancy was 14.3% (275 participants), among which anemia occurred most commonly during pregnancy (Table 1).

Table 2 showed the characteristics of the air pollutants and weather variables in Shanghai during the study period. The mean values of the daily average concentrations of SO_2_, NO_2_, and PM_10_ (23.7, 23.1, and 73.9 μg/m^3^, respectively) were lower than the limit values of China’s current air quality standards (limit values for Chinese urban areas: 150 μg/m^3^ for SO_2_, 80 μg/m^3^ for NO_2_ and 150 μg/m^3^ for PM_10_, respectively). The mean values of the daily average temperature, relative humidity, and wind speed were 21.5 °C, 70.6%, and 3.1 m/s, respectively. The ambient air pollutants were moderately correlated with one another (correlation coefficients range from 0.60 to 0.68) (Supplementary Table 1). The concentrations of air pollutants in the cool season were significantly higher than those in the warm season (*P* value < 0.05), and the PM_10_ concentrations increased much more compared with the changes of NO_2_ and SO_2_ at lag 0 day (Table 3).

**Figure 2:**
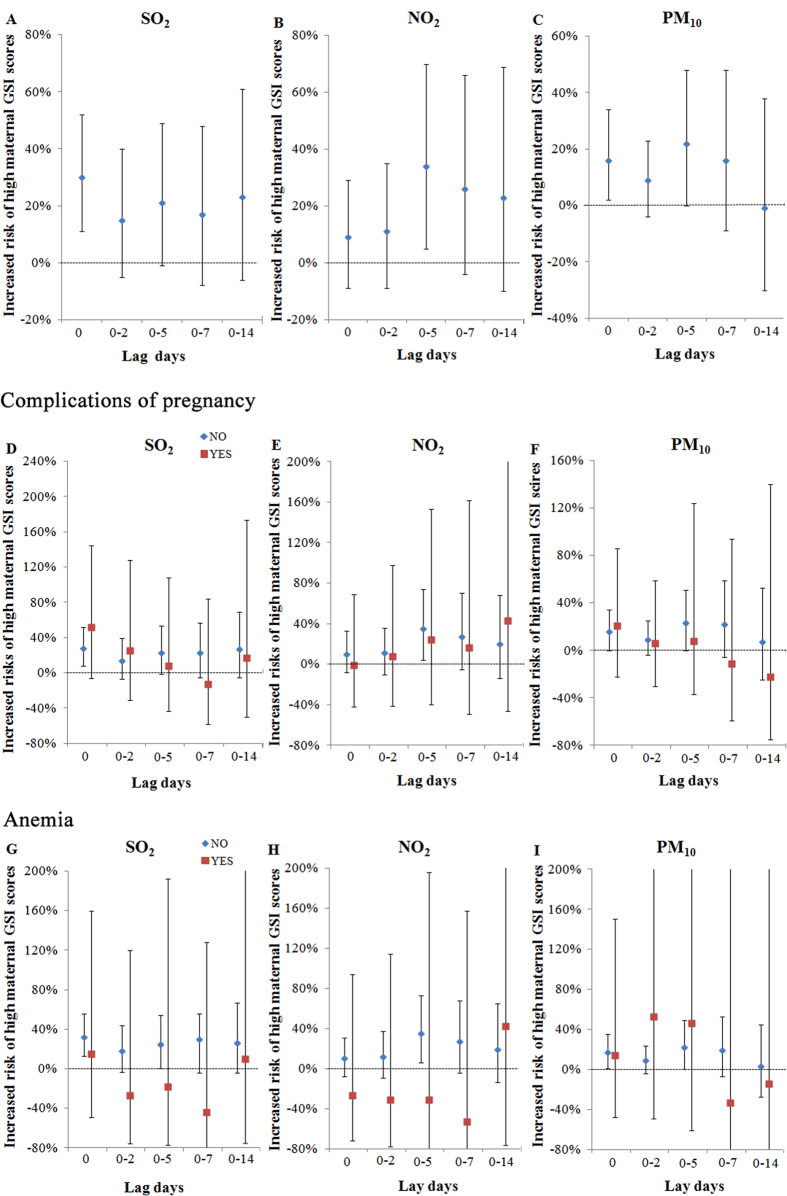
Increased risks of high maternal emotional stress (GSI scores: P75-P100) for an IQR increase in SO_2_ (A, IQR = 14.0 μg/m^3^), NO_2_ (B, IQR = 12.8 μg/m^3^), and PM_10_ (C, IQR = 57.0 μg/m^3^) at different lag days, and the modifying effects of complications of pregnancy (D–F) and anemia (G–I). The best fitting lag structures for SO_2_, NO_2_, and PM_10_ were lag 0 (**A**), lag 0–5 (**B**), and lag 0 (**C**), respectively. In **D–I**, blue square: women without complications of pregnancy/anemia; red square: women with complications of pregnancy/anemia. The association was stronger among participants without complications of pregnancy/anemia than among women with complications of pregnancy/anemia.

Figure 2A–C displayed the estimated risks of high maternal emotional stress (GSI among P75-P100) during pregnancy per IQR increase in air pollutant levels for different lag days. For high emotional stress, the best fitting lag structure for SO_2_, NO_2_, and PM_10_ was lag 0, lag 0–5, and lag 0, respectively. The per IQR increase in SO_2_ [OR = 1.30; 95% confidence intervals (CI): 1.11, 1.52; *P = *0.001] (Fig. 2A, Supplementary Table 2) and PM_10_ (OR = 1.16; 95% CI: 1.02, 1.34; *P = *0.029) (Fig. 2C, Supplementary Table 2) at lag 0 days and the per IQR increase in NO_2_ (Fig. 2B, Supplementary Table 2) at lag 0–5 days (OR = 1.34; 95% CI: 1.05, 1.70; *P = *0.018) were significantly associated with high maternal GSI during pregnancy. However, a null association between these three air pollutants and high GSI was found for the other lag day lengths.

After stratified by levels of air pollutants, we observed that the association of air pollution with the risk of maternal emotional stress was more evident among the high air pollution groups (P25-P100) than among the low pollution groups (P1-P25) (Table 4). We found that, among the high pollution groups (P25-P100), an IQR increase in SO_2_ concentrations at lag 0 day (OR = 1.36, 95%CI: 1.12–1.66), in PM_10_ concentrations at lag 0 day (OR = 1.19, 95%CI: 1.02–1.40) and lag 0–5 days (OR = 1.30, 95%CI: 1.04–1.62), and an IQR increase in NO_2_ concentrations at lag 0–5 days (OR = 1.60, 95%CI: 1.12–2.27) and lag 0–7 days (OR = 1.71, 95%CI: 1.13–2.59) were associated with increased risks of high maternal emotion stress (GSI among P75-P100) (all *P* value < 0.05). However, no significant association was found among the low pollution groups (P1-P25) (*P* > 0.05) (Table 4).

The association of air pollution with maternal stress changed with the season (Table 5). The association was much stronger in the cool season than in the warm season. The odds ratio of the association of PM_10_ with high maternal stress was higher and more significant in the cool season than in the warm season. However, we did not find any significant seasonal difference in the associations of SO_2_ and NO_2_ with high maternal GSI.

Figure 2D–F showed that the association between air pollution and maternal emotional stress was modified by complications of pregnancy. The association was stronger among the participants without complications of pregnancy than among the women with complications (Fig. 2D–F, Supplementary Table 2). Figure 2G–I showed that participants without anemia were more susceptible to air pollution exposures than those with anemia (Fig. 2G–I, Supplementary Table 2).

In two- and three-pollutant models, the inclusion of NO_2_ and PM_10_ into the models did not significantly influence the estimates for SO_2_ association with high GSI. However, the association of NO_2_ with maternal high GSI became non-significant when SO_2_ was added into the models. The association of PM_10_ with maternal high GSI became non-significant, and the estimates for the association decreased dramatically when SO_2_ was added into the model (Table 6).

Table 7 depicted the associations of maternal depression symptoms with SO_2_, NO_2_, and PM_10_. We observed that per IQR increase in SO_2_ at lag 0 day was significantly associated with high maternal depression symptom scores (OR = 1.22; 95% CI: 1.05, 1.42; *P* = 0.010). However, we found no significant associations of NO_2_ and PM_10_ with the high depression symptom scores.

In addition, the results revealed no significant association of each air pollutant with anxiety symptom scores and with the LESPW total scores (all *P* values > 0.05).

## Discussion

The present study is the first population-based study that focused on the association between air pollution and psychological health among pregnant women. Our results showed that increasing concentrations of SO_2_, NO_2_, and PM_10_ were all significantly associated with high emotional stress during pregnancy. The magnitude of the association may vary depending on the levels of air pollution, and may be modified by season and complications of pregnancy. Acute stress may be induced by exposure to SO_2_ and PM_10_, whereas a lagged stress may be a result of exposure to NO_2_.

Although the association between air pollution and stress has not been reported among pregnant women, previous studies that examined the adverse effects of air pollution on psychological wellbeing among old people were consistent with our study^35^. Bullinger found that increasing SO_2_ concentrations had adverse effects on mood and stress among females^36^. Szyszkowicz reported that SO_2_ and NO_2_ were associated with increased emergency department visits because of depression symptoms^14^. Air pollution, such as gases from the combustion of fossil fuels in motor vehicles, was reported to be associated with affective disorders and suicide attempts^16^. Animal research also indicated that air pollution could provoke depressive-like behaviours in mice^37^.

Currently, as far as we know, only one study has investigated the lag effect of air pollution on human psychological health^12^. Inconsistent with our study, the previous study found that the symptom of depression in the elderly were associated with a 3-day moving average concentration of PM_10_ and an 8-day moving average concentration of NO_2_, and a null association was found between SO_2_ and the symptom of depression^12^. We speculate that the difference between that study and ours may be due to the difference in air pollution levels. Additionally, the susceptibility to air pollutants may be different among different populations^38^.

The biological mechanisms underlying the effects of air pollution on maternal stress during pregnancy remain unknown. There were several possible explanations. First, air pollution during pregnancy may induce oxidative stress^39^. Oxidative stress has been implicated as the molecular mechanism in the pathogenesis of depression^40^. Pregnant women were reported to be much more vulnerable to oxidative stress than the general population because of their altered physiology and increased energy requirement^41^,^42^. Second, animal studies in mice have demonstrated that exposure to air pollution could induce the activation of the hypothalamic-pituitary-adrenal axis^43^, which was associated with stress^44^, and stress-related disorders^45^. Third, exposure to particulates was demonstrated to be associated with hippocampal pro-inflammatory cytokine expression and changes in hippocampal neuron morphology^37^, by which the neurotoxic effects of environmental pollutants on maternal stress may be mediated^13^,^46^.

We found that the association between air pollution and maternal stress during pregnancy differed under different levels of air pollution, which may suggest that if the pollution level was low, and the population did not realize that it was a “polluted” day, they may not worry about the impacts of air quality on their health. The possible explanations for the positive dose-dependent association between air pollution and maternal stress may be as follows. First, the effect of air pollution on oxidative stress, a potential molecular mechanism underlying the effect of air pollution on human psychological health, was dose-dependent. One previous study proved that high levels of air pollution were associated with oxidative stress, whereas no significant association was found between lower levels of air pollution and oxidative stress^23^. Second, higher levels of air pollution may induce a greater effect on inflammatory cytokine expression and neuron morphology in the hippocampus, which were related with the development of maternal stress^37^.

Consistent with studies reporting the seasonal difference in the effects of air pollution on mortality^34^, we found a stronger association between air pollution and maternal emotional stress in the cool season than in the warm season. This finding may be explained in part by the following reasons. First, the difference may be due to the different air pollution levels between the cool season and the warm season (the air pollution levels in the cool season were significantly higher than that in the warm season in our study). Second, based on a previous investigation, Shanghai residents closed their windows and used air conditioners more frequently in the summer than they did in the winter^34^, thus, the chance of exposure to outdoor air pollutants in the warm season or summer may be less than in the cool season or winter. Third, pregnant women may be more likely to go outdoors in the cool season or winter than in the warm season or summer because Shanghai is usually sunny with low humidity in the cool season and rainy with high humidity in the warm season^34^. Therefore, the seasonal difference in the association may be a result of different patterns of exposure to air pollutants among different seasons. However, the seasonal difference may vary by location in different parts of the world because of different weather conditions and lifestyles^47^.

This study reported that the complications of pregnancy appeared to modify the effects of air pollution on maternal emotional stress. Air pollution was closely associated with high maternal emotional stress among participants without complications of pregnancy, whereas the association was weaker among women with complications of pregnancy. Our results were consistent with those from a study performed among elderly people, which found that a history of cardiovascular disease may modify the association between air pollutants and the symptom of depression, and the association was weaker among elderly people with cardiovascular disease than those without cardiovascular disease^12^. One possible explanation for this phenomenon may be that when women were suffering from complications of pregnancy, they might care less about air pollution. However, caution should be taken when interpreting the results because we did not examine the modifying effect of each type of complication during pregnancy.

Air pollutants cannot be selectively inhaled in real life, and all of the air pollutants may be the indicators of the same pollutant mixture according to the results of the correlation analysis^48^. The associations of NO_2_ and PM_10_ became weaker after adjusting for SO_2_, indicating that among these three pollutants, SO_2_ may have a relatively stronger effect on maternal emotional stress than NO_2_ and PM_10_. Therefore, SO_2_ may be an important component in the air pollution mixture in Shanghai, and has an independently adverse effect on human psychological health. However, until now, there is no consensus on the comparison of the relative effects of these three pollutants. Consistent with our results, some studies found that the impact of SO_2_ was greater than that of NO_2_ and PM_10_^47^, whereas other studies found that the effect of PM_10_ was more significant than that of SO_2_^49^. This inconsistency may be a result of the difference in local meteorological conditions and the demographic characteristics of different populations^47^.

This study examined the association of air pollution with maternal symptom of depression. Our results indicated that SO_2_ may have a stronger effect on maternal symptom of depression during pregnancy than NO_2_ or PM_10_. Our study may provide an evidence for policy making regarding the control or reduction of total SO_2_ emissions.

There are several limitations in this study. First, this study was conducted from February to October, 2010. In Shanghai, the cool season is usually from November to the next April; therefore, we missed the time from November to next January. Thus, the statistical power on the comparison of the seasonal difference in the associaion of air pollution with maternal stress may be limited (a total of 476 women were enrolled in cool season (from February to April), and 1455 women were enrolled in warm season (from May to October) in this study. Second, because SO_2_, NO_2_ and PM_10_ were the only three criteria pollutants routinely monitored in Shanghai during our study period, we failed to investigate the association of other pollutants including ozone and carbon monoxide with maternal stress during pregnancy. Third, the noise data were not available in our study, and we were unable to control for the confounding effects of noise in our multivariable analyses. Fourth, the air pollution data were obtained from the monitoring stations of each district the women lived during pregnancy but were not based on each woman’s home. The distance between each woman’s home and the monitoring station, and the length of time each participant spent outdoors were not measured. Fifth, although we have tried our best to minimize the chance of selection bias in the sampling of study subjects, because we didn’t include all the pregnant women in Shanghai in this study, and considering the small number of districts in Shanghai using our stratified-cluster sampling approach, the possibility of selection bias cannot be completely excluded. Sixth, although extensive information on social-economic factors that may be potentially associated with maternal stress during pregnancy has been taken into consideration in our analyses, some factors that may explain the associations may not be included in this study.

There is no doubt that maternal stress is affected by more complex circumstances than simply air pollution. However, ambient air pollutants might be a triggering or exacerbating factor. Our findings may help elucidate the effects of air pollution on human psychological health. Because maternal stress during pregnancy is important in the development of offspring, further in-depth studies are required to bring these issues to the public’s attention. Further control of air pollution, particularly SO_2_, may result in health benefits.

## Conclusions

In conclusion, our study suggested that increasing concentrations of SO_2_, NO_2_, and PM_10_ were associated with increased risks of maternal emotional stress during pregnancy, and the associations between air pollution and maternal stress may be dose-dependent and may be modified by season and complications of pregnancy. Exposure to SO_2_ and PM_10_ may induce acute development of maternal emotional stress, whereas exposure to NO_2_ may cause a lagged stress. Increasing concentrations of SO_2_ may also be a risk factor for maternal depressive symptoms.

## Additional Information

**How to cite this article**: Lin, Y. *et al*. The impacts of air pollution on maternal stress during pregnancy. *Sci. Rep.* **7**, 40956; doi: 10.1038/srep40956 (2017).

**Publisher's note:** Springer Nature remains neutral with regard to jurisdictional claims in published maps and institutional affiliations.

## Supplementary Material

Supplementary Information

## Figures and Tables

**Table 1:** Socio-demographic characteristics of the 1,931 participants

Variable names	No (%), mean ± SD, or Medium (P25, P75)
**Age (years); missing n = 9**	28.0 (4.0)
**Maternal education; missing n = 8**	
Junior high school or lower	442 (23.0%)
Senior high school	353 (18.4%)
College or University level	985 (51.2%)
Postgraduate level	143 (7.4%)
**Family monthly income (RMB/month); missing n = 37**	
<2,000	311 (16.4%)
2,000–5,000	699 (36.9%)
5,000–10,000	565 (29.8%)
>10,000	319 (16.8%)
**Type of family structure; missing n = 17**	
Living with husband	997 (52.2%)
Living with husband and parents-in-law	605 (31.7%)
Living with parents	222 (11.6%)
Living alone	87 (4.6%)
**Complications of pregnancy; missing n = 3**	
No	1653 (85.7%)
Yes	275 (14.3%)
**Abortion history; missing n = 15**	
Never	1199 (62.6%)
1–2 times	654 (34.1%)
3 or more times	63 (3.3%)
**Maternal GSI score**	1.2 (1.1, 1.3)
**Total score of LESPW; missing n = 3**	230.7 (84.0, 332.0)

**Table 2:** Weather and air pollution levels in Shanghai during the study period.

	Mean ± SD	Median	Range	IQR
Mean temperature (°C)	21.5 ± 6.7	22.5	−0.6–34.1	17.2–26.8
Relative humidity (%)	70.6 ± 12.3	71.0	38.0–93.0	64.0–80.0
Wind speed (m/s)	3.1 ± 1.1	3.0	1.2–6.6	2.4–3.8
Mean SO_2_ (μg/m^3^)	23.7 ± 10.3	22.0	6.0–80.0	16.0–30.0
Mean NO_2_ (μg/m^3^)	23.1 ± 8.6	23.2	4.8–79.2	16.8–29.6
Mean PM_10_ (μg/m^3^)	73.9 ± 46.8	62.0	14.0–310.0	39.0–96.0

**Table 3:** Geometric means (95%CI) of air pollutant concentrations in different seasons.

Air pollutants	Lag days	Cool season (n = 476)	Warm season (n = 1455)	*P*-value a
SO_2_	Lag 0	22.19 (21.29, 23.14)	21.34 (20.87, 21.82)	0.141
	Lag 0–2	24.23 (23.55, 24.93)	21.40 (21.01, 21.79)	0.081
	Lag 0–5	25.41 (24.66, 26.19)	20.97 (20.63, 21.31)	0.002
	Lag 0–7	25.32 (24.64, 26.02)	21.24 (20.91, 21.57)	<0.001
	Lag 0–14	25.62 (25.02, 26.23)	21.83 (21.54, 22.12)	<0.001
NO_2_	Lag 0	22.15 (21.32, 23.01)	21.20 (20.79, 21.62)	<0.001
	Lag 0–2	24.57 (23.90, 25.27)	21.40 (21.05, 21.76)	<0.001
	Lag 0–5	24.67 (24.08, 25.28)	20.96 (20.66, 21.28)	<0.001
	Lag 0–7	24.06 (23.56, 24.57)	21.07 (20.76, 21.39)	<0.001
	Lag 0–14	23.77 (23.36, 24.19)	21.14 (20.87, 21.41)	<0.001
PM_10_	Lag 0	67.29 (63.37, 71.46)	60.30 (58.55, 62.10)	<0.001
	Lag 0–2	70.45 (66.62, 74.51)	61.61 (60.34, 62.91)	<0.001
	Lag 0–5	67.04 (64.16, 70.05)	59.73 (58.63, 60.85)	<0.001
	Lag 0–7	69.22 (66.41, 72.15)	61.38 (60.33, 62.44)	<0.001
	Lag 0–14	71.97 (69.18, 74.87)	63.72 (62.79, 64.67)	<0.001

^a^ANOVA was used to examine the difference of the means (log transformation) of air pollution concentrations between the cool season and the warm season.

**Table 4:** The dose-dependent association between levels of air pollutants (per IQR increase) and the risks of high maternal emotional stress^a^.

Air pollutants	The odds ratios (95%CI) of high maternal stress (GSI: P75-P100)				
	Lag 0	Lag 0–2	Lag 0–5	Lag 0–7	Lag 0–14
SO_2_ (μg/m^3^)					
<25 th	0.33 (0.06, 1.96)	0.46 (0.10, 2.07)	1.36 (0.24, 7.76)	2.16 (0.53, 8.85)	1.07 (0.28, 4.11)
≥25 th	1.36 (1.12, 1.66)*	1.12 (0.88, 1.44)	1.22 (0.92, 1.61)	1.22 (0.87, 1.71)	1.05 (0.71, 1.57)
NO_2_ (μg/m^3^)					
<25 th	1.13 (0.28, 4.50)	1.01 (0.32, 3.19)	0.54 (0.8, 1.59)	0.51 (0.19, 1.40)	1.34 (0.45, 3.95)
≥25 th	1.04 (0.82, 1.32)	1.00 (0.76, 1.32)	1.60 (1.12, 2.27)*	1.71 (1.13, 2.59)*	1.35 (0.83, 2.19)
PM_10_ (μg/m^3^)					
<25 th	3.93 (0.40, 38.18)	0.17 (0.02, 1.29)	3.77 (0.31, 45.77)	0.94 (0.06, 13.92)	2.02 (0.10, 42.59)
≥25 th	1.19 (1.02, 1.40)*	1.09 (0.95, 1.26)	1.30 (1.04, 1.62)*	1.23 (0.93, 1.63)	1.14 (0.76, 1.70)

^*^P < 0.05.

^a^Adjusted for maternal age, education, occupation, complications of pregnancy, abortion history, family monthly income, structues of family type, average temperature, relative humidity, and wind speed.

**Table 5:** Increased risks [OR (95%CI)] of high maternal emotional stress (GSI: P75-P100) for an IQR increase in SO_2_, NO_2_, and PM_10_ concentrations by season^a^.

Air pollutants	Lag days	Cool season^b^	Warm season^c^
SO_2_	Lag 0	1.31 (1.00, 1.73)	1.21 (0.98, 1.49)
	Lag 0–2	1.21 (0.84, 1.75)	0.99 (0.78, 1.27)
	Lag 0–5	1.19 (0.82, 1.73)	1.11 (0.84, 1.45)
	Lag 0–7	0.93 (0.56, 1.55)	1.06 (0.79, 1.44)
	Lag 0–14	1.06 (0.58, 1.94)	1.12 (0.79, 1.58)
NO_2_	Lag 0	1.05 (0.78, 1.42)	1.11 (0.89, 1.39)
	Lag 0–2	1.14 (0.79, 1.63)	1.04 (0.81, 1.34)
	Lag 0–5	1.58 (0.98, 2.54)	1.29 (0.94, 1.78)
	Lag 0–7	1.37 (0.77, 2.43)	1.17 (0.82, 1.66)
	Lag 0–14	1.30 (0.67, 2.54)	1.20 (0.80, 1.79)
PM_10_	Lag 0	1.28 (1.04, 1.57)*	1.04 (0.86, 1.27)
	Lag 0–2	1.09 (0.93, 1.27)	1.00 (0.75, 1.33)
	Lag 0–5	1.25 (0.91, 1.72)	1.19 (0.89, 1.61)
	Lag 0–7	1.06 (0.62, 1.82)	1.08 (0.76, 1.55)
	Lag 0–14	0.68 (0.34, 1.37)	1.14 (0.56, 2.31)

^a^Adjusted for maternal age, education, occupation, complications of pregnancy, abortion history, family monthly income, types of family structure, average temperature, relative humidity, and wind speed.

^b^Cool season: from November to the next April.

^c^Warm season: from May to October.

^*^The risks were significantly different between the warm season and the cool season (*P *<0.05).

**Table 6:** Air pollutant levels (an IQR increase) and increased risks of maternal high emotional stress (GSI: P75-P100) using single-, two-, and three pollutant models.

Pollutants and models	Lag days	OR (95% CI)	*P*-value
SO_2_			
Single model^a^	Lag 0	1.30 (1.11, 1.52)*	0.001
+NO_2_	Lag 0	1.38 (1.14, 1.66)*	0.001
+PM_10_	Lag 0	1.27 (1.05, 1.53)*	0.013
+NO_2_+PM_10_	Lag 0	1.33 (1.09, 1.63)*	0.006
NO_2_			
Single model^a^	Lag 0–5	1.34 (1.05, 1.70)*	0.018
+SO_2_	Lag 0–5	1.28 (0.94, 1.75)	0.116
+PM_10_	Lag 0–5	1.25 (0.95, 1.65)	0.111
+SO_2_+PM_10_	Lag 0–5	1.24 (0.90, 1.71)	0.195
PM_10_			
Single model^a^	Lag 0	1.16 (1.02, 1.34)*	0.029
+SO_2_	Lag 0	1.04 (0.88, 1.23)	0.640
+NO_2_	Lag 0	1.18 (1.00, 1.38)	0.054
+SO_2_+PM_10_	Lag 0	1.08 (0.91, 1.29)	0.382

^*^P < 0.05.

^a^Adjusted for maternal age, education, occupation, complications of pregnancy, abortion history, family monthly income, types of family structure, average temperature, relative humidity, and wind speed.

**Table 7:** Estimated effects of air pollutants (per IQR increase) on high maternal depression scores (P75-P100)a, b.

Item	Lag day	SO_2_ (IQR = 14.0 μg/m^3^)	NO_2_ (IQR = 12.8 μg/m^3^)	PM_10_ (IQR = 57.0 μg/m^3^)
		OR (95% CI)	OR (95% CI)	OR (95% CI)
Depression scores	Lag 0	1.22 (1.05, 1.42)*	1.05 (0.89, 1.24)	1.08 (0.94, 1.23)
	Lag 0–2	1.11 (0.92, 1.33)	1.02 (0.84, 1.23)	1.04 (0.92, 1.18)
	Lag 0–5	1.12 (0.92, 1.37)	1.21 (0.96, 1.52)	1.13 (0.94, 1.37)
	Lag 0–7	1.10 (0.88, 1.38)	1.19 (0.92, 1.54)	1.09 (0.86, 1.38)
	Lag 0–14	1.15 (0.89, 1.49)	1.20 (0.89, 1.63)	1.05 (0.76, 1.45)

^*^P < 0.05.

^a^Logistic regression models were used.

^b^Adjusted for maternal age, education, occupation, complications of pregnancy, abortion history, family monthly income, types of family structure, average temperature, relative humidity, and wind speed.
