# Supplementary material for: The impacts of air pollution on maternal stress during pregnancy
Source: Sci Rep. 2017 Jan 18;7:40956. doi: 10.1038/srep40956 (PMC5241869; doi:10.1038/srep40956)
Supplement: Supplementary Information [file srep40956-s1.pdf]

Supplementary material

The impacts of air pollution on maternal stress during pregnancy

Yanfen Lin[1†], Leilei Zhou[1†], Jian Xu[1,*] , Zhongcheng Luo[1], Haidong Kan[2,3], Jinsong Zhang[1],

Chonghuai Yan[1], Jun Zhang[1]

[1] Xinhua Hospital, MOE-Shanghai Key Laboratory of Children's Environmental Health, Department of Child and Adolescent Healthcare, Shanghai Institute for Pediatric Research, Shanghai Jiao Tong University School of Medicine, Shanghai, 200092 China

[2] School of Public Health, Key Laboratory of Public Health Safety of the Ministry of Education and Key Laboratory of Health Technology Assessment of the Ministry of Health, Fudan University, Shanghai, 200032 China

[3] Shanghai Key Laboratory of Atmospheric Particle Pollution and Prevention (LAP[3]), Fudan University, Shanghai, 200032 China

*Corresponding author: Xinhua Hospital, MOE-Shanghai Key Laboratory of Children's Environmental Health, Department of Child and Adolescent Healthcare, Shanghai Institute for Pediatric Research, Shanghai Jiao Tong University School of Medicine, Shanghai, 200092 China. Address: No. 1665 Kongjiang Road, Shanghai, China. Tel: 86-21-25078864. Fax: 86-21-25078875. Email address: sonia0616@sjtu.edu.cn (Jian Xu).

[†]These two authors contributed equally to this work.

Supplementary Table 1. Spearman correlation coefficients between air pollutants and weather variables in this study

	Relative humidity	Wind speed	SO_2	NO_2	PM_{10}
Temperature	-0.02	0.27**	0.17**	-0.01	0.03
Relative humidity		-0.06**	-0.34**	-0.06*	-0.34**
Wind speed			-0.28**	-0.44**	-0.33**
SO_2				0.60**	0.68**
NO_2					0.65**

**$P<0.01$

*$P<0.05$

Supplementary Table 2. Increased risk [OR (95%CI)] of high maternal emotional stress (GSI: P75-P100) for an IQR increase in SO_2, NO_2, and PM_{10}, and the modifying effect of complications of pregnancy[a]

**Lag days**	**SO_2**	**NO_2**	**PM_{10}**
**Lag 0**			
Total	**1.30 (1.11, 1.52)***	1.09 (0.91, 1.29)	**1.16 (1.02, 1.34)***
With complications of pregnancy	1.52 (0.94, 2.44)	0.99 (0.58, 1.69)	1.21 (0.78,1.86)
Without complications of pregnancy	**1.28 (1.08, 1.52)***	1.10 (0.92, 1.33)	1.16 (1.00, 1.34)
**Lag 0-2**			
Total	1.15 (0.95, 1.40)	1.11 (0.91, 1.35)	1.09 (0.96, 1.23)
With complications of pregnancy	1.25 (0.69, 2.28)	1.08 (0.59, 1.98)	1.06 (0.70, 1.59)
Without complications of pregnancy	1.14 (0.93, 1.39)	1.11 (0.90, 1.36)	1.09 (0.96, 1.25)
**Lag 0-5**			
Total	1.21 (0.99, 1.49)	**1.34 (1.05, 1.70)***	1.22 (1.00, 1.48)
With complications of pregnancy	1.08 (0.57, 2.08)	1.24 (0.60, 2.53)	1.19 (0.63, 2.24)
Without complications of pregnancy	1.23 (0.99, 1.53)	**1.35 (1.04, 1.74)***	1.23 (1.00, 1.51)
**Lag 0-7**			
Total	1.17 (0.92, 1.48)	1.26 (0.96, 1.66)	1.16 (0.91, 1.48)
With complications of pregnancy	0.87 (0.42, 1.84)	1.16 (0.51, 2.62)	0.89 (0.41, 1.94)
Without complications of pregnancy	1.23 (0.95, 1.57)	1.27 (0.95, 1.70)	1.22 (0.94, 1.59)
**Lag 0-14**			
Total	1.23 (0.94, 1.61)	1.23 (0.90, 1.69)	0.99 (0.70, 1.38)
With complications of pregnancy	1.17 (0.50, 2.73)	1.43 (0.54, 3.82)	0.78 (0.25, 2.40)
Without complications of pregnancy	1.27 (0.95, 1.69)	1.20 (0.86, 1.68)	1.07 (0.75, 1.53)

*$P<0.05$

[a] Adjusted for maternal age, education, occupation, complications of pregnancy, abortion history, family monthly income, type of family structure, average temperature, relative humidity, and wind speed.
